# Cryopreservation of human mesenchymal stromal cells expressing TRAIL for human anti-cancer therapy

**DOI:** 10.1016/j.jcyt.2016.04.005

**Published:** 2016-07

**Authors:** Zhengqiang Yuan, Sofia Da Silva Lourenco, Elizabeth K. Sage, Krishna K. Kolluri, Mark W. Lowdell, Sam M. Janes

**Affiliations:** 1 Lungs for Living Research Centre, UCL Respiratory, Division of Medicine, University College London, London, United Kingdom; 2 Centre for Cell, Gene & Tissue Therapy, Royal Free London National Health Services Foundation Trust & University College London, London, United Kingdom

**Keywords:** apoptosis, chemokine, cryopreservation, DMSO, MSC, ZENALB 4.5

## Abstract

**Background aims:**

Mesenchymal stromal cells (MSCs) are being extensively researched for cell therapy and tissue engineering. We have engineered MSCs to express the pro-apoptotic protein tumor necrosis factor–related apoptosis inducing ligand (TRAIL) and are currently preparing this genetically modified cell therapy for a phase 1/2a clinical trial in patients with metastatic lung cancer. To do this, we need to prepare a cryopreserved allogeneic MSCTRAIL cell bank for further expansion before patient delivery. The effects of cryopreservation on a genetically modified cell therapy product have not been clearly determined.

**Methods:**

We tested different concentrations of dimethyl sulfoxide (DMSO) added to the human serum albumin ZENALB 4.5 and measured post-thaw cell viability, proliferation ability and differentiation characteristics. In addition, we examined the homing ability, TRAIL expression and cancer cell–killing capacities of cryopreserved genetically modified MSCs compared with fresh, continually cultured cells.

**Results:**

We demonstrated that the post-thaw viability of MSCs in 5% DMSO (v/v) with 95% ZENALB 4.5 (v/v) is 85.7 ± 0.4%, which is comparable to that in conventional freezing media. We show that cryopreservation does not affect the long-term expression of TRAIL and that cryopreserved TRAIL-expressing MSCs exhibit similar levels of homing and, importantly, retain their potency in triggering cancer cell death.

**Conclusions:**

This study shows that cryopreservation is unlikely to affect the therapeutic properties of MSCTRAIL and supports the generation of a cryopreserved master cell bank.

## Introduction

Mesenchymal stromal cells (MSCs) possess a number of unique properties that make them attractive candidates for cellular based therapies. They are readily isolated from multiple adult and neonatal tissues and are easy to expand in *ex vivo* culture conditions [1]. They secrete a wide range of soluble growth factors and cytokines that can be immunomodulatory, anti-apoptotic, anti-inflammatory and anti-fibrotic and can stimulate repair and regeneration at the site of tissue injury [2]. In addition to being attracted to sites of injury, they show evidence of tumor tropism and incorporation into the tumour microenvironment [3], making them ideal vehicles for the delivery of targeted anti-cancer therapies using both systemic and topical delivery.

These properties have been harnessed further by genetic modification of MSCs using integrating vectors [4], resulting in long-term stable gene expression without affecting the cells critical characteristics [5], [6]. Several groups have combined the characteristics of tumor tropism and long-term genetic modification to develop targeted anti-cancer therapies [7], [8], [9], [10], [11]. In addition, it seems that MSCs are immunologically inert because of their low expression of constitutive major histocompatibility complex 1 (MHC1) and lack of MHC2 and co-stimulatory molecules CD80, CD86 and CD40, meaning that allogeneic cells can be used without the need for immunosuppressive therapy in the recipient [12].

Thus, it is no surprise that there is great interest in the development of gene and cellular therapies for the clinic. There are currently more than 500 clinical trials testing MSCs as therapies for a wide range of diseases, and of these more than 35% are using cryopreserved cells. From a commercial perspective, the use of cryopreserved cells has significant advantages over fresh cells, including quality control, standardization of product and the production of an immediate off-the-shelf therapeutic supply to allow better timing of therapy. In addition, it is essential to cryopreserve MSCs at an early passage because many of their properties decrease with increasing passage.

We have developed a novel targeted genetically modified MSC therapy for metastatic lung cancer [7], [11], [13] and malignant mesothelioma [9] that is undergoing preparation for delivery in a phase 1/2a clinical trial to patients with metastatic lung cancer. The first step is the preparation of a master cell bank of allogeneic MSCs transduced with a lentiviral vector expressing tumor necrosis factor–related apoptosis inducing ligand (MSCTRAIL) that will be expanded to produce a working cell bank and cryopreserved in a desired concentration until required for delivery to patients. Cryopreserved allogeneic MSCs have been used in many previous clinical trials in the treatment of respiratory disease [14] but more widely in graft-versus-host disease [15], [16], [17] and in cardiac disease for the treatment of acute myocardial infarction [18] and ischemic cardiomyopathy [19]. Despite significant evidence of a positive safety profile of these cells, from an efficacy perspective the trial results have been disappointing with limited therapeutic improvement. One reason proposed for the lack of clinical efficacy seen in patients is that cryopreservation of MSCs results in both apoptosis of cells on thawing and a reduction in *in vitro* potency compared with continuously cultured cells [20], [21], [22]. To date the majority of clinical trials using MSCs are doing so to exploit their immunomodulatory properties, and it has been these properties that are effected post-cryopreservation. Our clinical trial is the first to exploit the tumor tropic characteristics of MSCs along with long-term gene expression, and the effects of cryopreservation on these has not been assessed.

MSCs for clinical use are commonly frozen in 5–10% dimethyl sulfoxide (DMSO) and fetal bovine serum (FBS) [2], [23], [24], but there are disadvantages of using these agents. DMSO is toxic at high concentrations and has been reported to cause adverse events in patients [25], [26], and the use of animal proteins theoretically risks transmitting infectious agents or stimulating immunological responses. ZENALB 4.5 is a protein supplement obtained from human plasma that is already in clinical use and would be a suitable replacement for FBS. In this study, we show that MSCs can be cryopreserved in 5% DMSO with 95% ZENALB4.5 without a significant adverse effect on cell viability, and those cells can be left for up to 90 min post-thaw without adversely affecting viability. We also demonstrate for the first time that cryopreservation does not affect long-term expression of TRAIL and that there is a minimal reduction in migration of thawed cells compared with fresh cells. Finally, and most importantly, our thawed cells retain their potency in causing cancer cell death.

## Methods

### Chemicals

All chemicals used in this study were purchased from the Sigma (UK) and culture medium from Invitrogen.

### Cell culture

Well-characterized human bone marrow–derived MSCs (passage 1) were purchased from the Texas A&M Health Science Center and cultured in α-Minimum Essential Medium (MEM) containing 17% FBS, 200 units/mL penicillin and streptomycin and L-glutamine. MSCs were transduced with lentivirus expressing full length TRAIL as previously described [13]. The culture medium for MSCs was changed twice a week. Passage 4 MSC and MSCTRAIL cells were cultured and harvested at ~80% confluence using TrypLE (A12859, Life Technologies) for dissociation and centrifuged. The harvested passage 5 cells were used for cryopreservation studies or other assays. Two cancer cell lines A549 and MDAMB231 (M231) were tested. M231 is a human breast adenocarcinoma line and A549 is a human lung adenocarcinoma line. M231 is sensitive to recombinant TRAIL (rTRAIL) treatment, whereas A549 is highly resistant. Both A549 and M231 were obtained from Cancer Research UK and grown in Dulbecco's Modified Eagle's Medium containing 10% FBS.

### Cryopreservation of MSCs

The previously used routine freezing solution (5% DMSO, 30% FBS in alpha-MEM medium (Gibco-BRL) was used as a control for cryopreservation study. The tested freezing solutions were prepared in a human albumin 4.5% solution (ZENALB 4.5, Bio Products Laboratory) containing either no DMSO (D0) or increasing concentrations of DMSO (CryoSure-DMSO, WAK-Chemie Medical) from 0.5% to 20% (D0.5–D20, respectively). Cells were harvested, washed with 1 × phosphate-buffered saline, then cell pellets directly resuspended in 1 mL of freezing solution at concentrations of 1 × 10^6^ cells/mL, 5 × 10^6^ cells/mL or 10 × 10^6^ cells/mL, transferred into cryovials followed by placing the cryovials in an isopropanol freezing box (Nalgene cryo 1°C/min freezing container, Nalgene) for overnight freezing in a −80°C freezer (New Brunswick Scientific), and then stored in liquid nitrogen vapour (Taylor-Wharton) for at least 1 week and up to 3 weeks. Before use, the cells were thawed by rapidly immersing the cryovials in a 37°C water bath with gentle shaking for 2 min, followed by transferring cells into 9 mL of warmed α-MEM for wash and cells pelleted by centrifugation at 1100 rpm for 5 min. The pelleted cells were then suspended in apoptosis assay solution for cell viability assessment or suspended in culture medium for functionality analyses.

### Cell viability and apoptosis assay

To examine cell viability and apoptosis, cells were defrosted as described earlier and left in cell suspension for 0–150 min, then stained with annexin V-AF647 antibody (Invitrogen) and 4′, 6-diamidino-2-phenylindole (DAPI; 200 µg/mL; Sigma) and assessed by flow cytometry. Annexin V+ cells were considered to have undergone apoptosis; Annexin V+/DAPI+ cells were considered to be dead by apoptosis; Annexin V-/DAPI-cells were considered to be viable.

### Co-culture and apoptosis analysis

A549 and M231 cancer cells were labeled with DiI as previously described [9], 8000 cells were plated into each well of a 96-well plate and 2000 fresh or cryopreserved MSCs, or control medium was added and left for 24 h. Floating and adherent cells were collected, stained with annexin V-AF647 and 2 µg/mL DAPI (Sigma) and assessed by flow cytometry. As before, annexin V+ cells were considered to have undergone apoptosis; Annexin V+/DAPI+ cells were considered to be dead by apoptosis.

### Proliferation assay of cryopreserved MSCs

Passage 5 MSCs were thawed, seeded at a density of 10 000 cells per well of a 24-well plate and left to grow for 6 days. Assessment of cell proliferation was determined every 72 h using the XTT Cell Proliferation Assay Kit according to the manufacturer's instructions.

### MSC phenotyping and differentiation assay

Cryopreserved MSCs were thawed, washed and cultured for 3 days before phenotyping by using the human MSC Phenotyping Kit (Miltenyi Biotec, Cat. No. 130-095-198) according to the manufacturer's instructions. Differentiation of cryopreserved MSCs was performed by using the StemPro Chondrogenesis, Osteogenesis or Adipogenesis Differentiation Kits (GIBCO Invitrogen Cell Culture). Adipocytes were stained with HCS LipidTOX TM Green and DAPI, osteocytes were stained with alizarin red S and the chondrogenic pellet was stained with alcian blue, all according to the manufacturer's instructions. MSCs cryopreserved by control solution (Cont) and by D5 solution (D5) were both examined for a comparison of marker protein expression.

### Migration assay

MSC migration capacity was assessed using Transwell plates (24-well plate format; BD Biosciences) and 8-µm inserts. The inserts were coated with pure collagen (BD Biosciences) before seeding 1 × 10^4^ MSCs in 100 µL per well. The lower chamber was filled with 600 µL of MDAMB231 conditioned medium as a chemotactic solution. Cells that migrated to the bottom of the insert were fixed with 4% paraformaldehyde, stained with DAPI and counted manually using a fluorescence microscope (Axioskop2; Zeiss).

### Flow cytometry of lentivirus-transduced cells

To determine the effects of cryopreservation on cell viability, MSCs were stained with annexin V-AF647 and 2 µg/mL DAPI (Sigma) and then assessed by flow cytometry. For the expression detection of TRAIL or CXCR4, MSC cells were stained with a phycoerythrin (PE)-conjugated mouse mAb (1:10 dilution) against human TRAIL (Ab47230, Abcam) or a PE anti-human CXCR4 mAb (Cat. 306506, Biolegend; 1:10 dilution), respectively, and analysed by flow cytometry.

### Statistical analysis

Data were analyzed with the use of GraphPad Prism 6 software (GraphPad Software) and presented as mean ± SD of at least three separate experiments. Statistical significance between groups was determined by use of the Student's *t* test for post-thaw cell viability and cancer cell co-culture assessment or Bonferroni multiple comparison statistic test for migration assay. Significant probability values are denoted as *P <* *0.05; **P <* *0.01; ***P <* *0.001.

## Results

### Cryopreservation of MSCs

**Figure 1:**
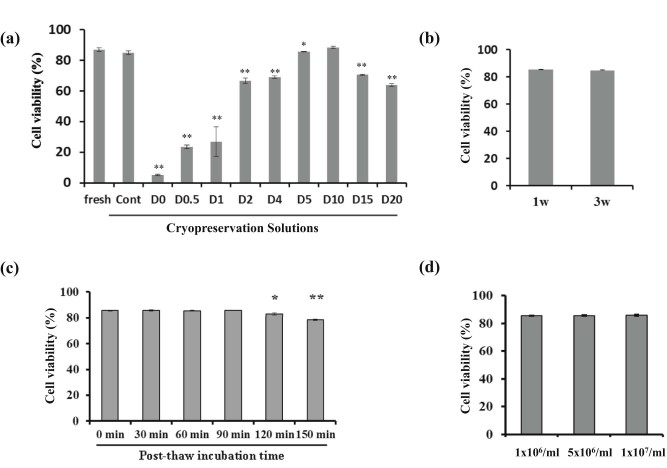
Assessment of post-thaw MSCTRAIL viability by apoptosis assay. (a) Cell viability was determined on continuously cultured fresh cells and post-thaw cryopreserved cells. Fresh, continuously cultured fresh cells; Cont, cells cryopreserved for 1 week in control routine freezing solution; D0–D20, cells cryopreserved for 1 week in increasing concentrations of DMSO in 4.5% human albumin 4.5% solution. Data were presented as mean ± SD of at least three separate experiments. *P <* *0.05, **P <* *0.01 compared with Cont, respectively, by Student's *t*-test. (b) Long-term storage cell viability. MSCTRAILs, cryopreserved in D5 solution for 1 week (1w) or 3 weeks (3w), were analyzed. (c) MSCTRAILs were cryopreserved in D5 for 1 week, then post-thaw cell viability was determined immediately after thawing (0 min) or after being left in the thawed D5 solution for 30, 60, 90, 120 or 150 min. *P <* *0.05, **P <* *0.01 compared with viability at 0 min, respectively, analyzed by Student's *t*-test. (d) Post-thaw cell viability for various cryopreservation densities as indicated. Cells were frozen for 1 week. Cell viability was measured by flow cytometry using AF647-annexin V/DAPI staining. Cells negative for both annexin V and DAPI were regarded as viable cells.

To determine the effects of different cryopreservant combinations on MSC viability cell freezing solutions were prepared as described in Table I. Cell viability after thawing was assessed by annexin V and DAPI staining using flow cytometry. Cell viability of continuously cultured fresh MSCTRAIL was 87.05 ± 1.20% and this was maintained in standard control cryopreservation media containing 30% FBS, which gave 85.07 ± 1.25% post-thaw cell viability (Figure 1A). Compared with fresh cells, the viability of MSCTRAIL cryopreserved in DMSO-free solution D0 was significantly reduced to 5.16 ± 0.54% only. Increasing concentrations of DMSO shows an increase in cell viability compared with no DMSO, upto a concentration of 10%. Cell viability in 10% DMSO and 90% ZENALB 4.5 (88.5 ± 0.71%) was similar to that of continuously cultured fresh counterpart (87.05 ± 1.02%) and slightly better than in control cryopreservation solution Cont (85.07 ± 1.25%; Figure 1a). From a clinical perspective, the total concentration of DMSO delivered to patients is important because high DMSO concentrations can result in adverse reactions including nausea, headache, hypotension and gastrointestinal upset [25], [26]. To reduce this effect, it is important to keep the concentration of DMSO to a minimum, and our results show that although there is a slight reduction in cell viability using 5% DMSO (85.65 ± 0.35%) compared with using 10% DMSO containing solution D10 (88.5 ± 0.71%), this would be worth tolerating to enable a reduction in DMSO delivery to the patient. Of note, >10% of DMSO included in the ZENALB 4.5 solution, that is, 15% and 20% DMSO-containing freezing solutions D15 and D20, cause a significant reduction in MSC viability (70.6 ± 0.42% and 64.1 ± 0.85%, respectively).

Because 5% DMSO in ZENALB 4.5 freezing solution has shown a compatible cell viability preservation with those frozen in control formulation, this will be used in the clinical trial setting. For this reason, all subsequent cells tested were frozen in the D5 freezing solution. Long-term cryopreservation storage of MSCTRAIL cells showed that MSCs cryopreserved in D5 solution showed no change in post-thaw cell viability for up to at least 3 weeks (Figure 1b).

In a clinical setting, cryopreserved MSCs will be rapidly thawed at the patient's bedside and immediately delivered by intravenous administration. It is therefore important to demonstrate the stability of MSCTRAIL in the cryopreservation media. We examined post-thaw MSCTRAIL viability after different incubation periods ranging from 0 to 150 min by apoptosis assay. Cell viability was 85.65 ± 0.35% immediately post-thaw, and this viability was maintained for up to 90 min (Figure 1c). At 120 min, cells started to show a significant fall in viability (82.7 ± 0.85%), which dropped further when left for 150 min (78.5 ± 0.57). This is relevant because it gives an indication of the stability of the post-thaw product and will ensure that protocols can be designed to ensure that infusion of cells occurs within 90 min.

A final consideration regarding an “off-the-shelf” cryopreserved cell therapy product is the cell density at which it can be frozen. We tested cell viability of MSCTRAIL after cryopreservation at cell densities of 1 × 10^6^, 5 × 10^6^ and 1 × 10^7^ and demonstrated no significant difference in post-thaw viabilities of approximately 85% (Figure 1d).

### Proliferation, morphology and differentiation of cryopreserved MSCTRAIL

**Figure 2:**
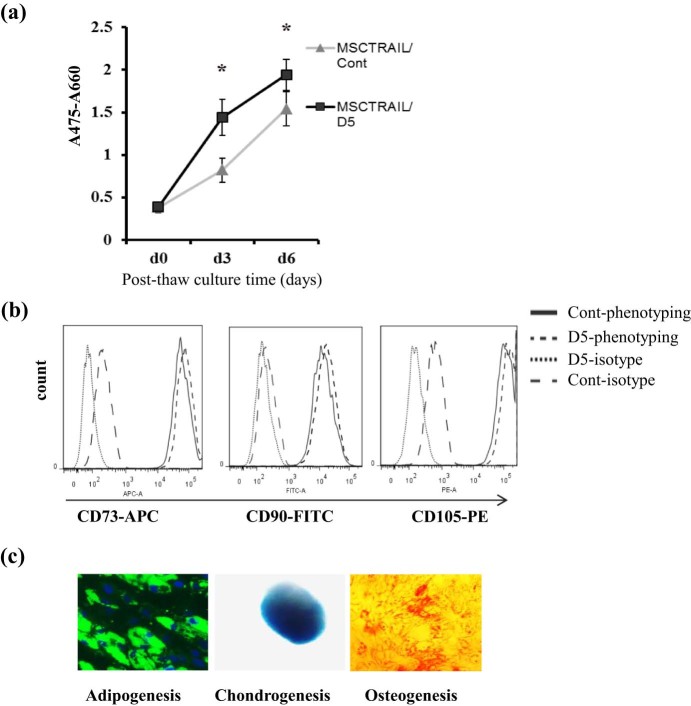
Cryopreservation does not affect MSC proliferation, marker protein expression and differentiation potential. (a) Post-thaw cell viability and proliferation were assessed using the XTT assay for 6 days. (b) Phenotyping of cryopreserved MSCTRAIL by detection of MSC markers. Cryopreserved cells were thawed and cultured for 3 days before phenotyping analyses. (c) Post-thaw MSCTRAIL differentiation capacity was assessed by culturing thawed cells in adipogenic, chondrogenic or osteogenic differentiation media. Left, HCS LipidTOX Green staining (green) for neutral lipid and DAPI staining for nuclei (blue) to show adipogenic differentiation; middle, alcian blue staining (blue) to show chondrogenic differentiation; and right, alizarin red S staining (red) to show osteogenic differentiation. Magnification ×200 for adipogenesis assay, ×100 for osteogenesis assay and ×50 for chondrogenesis assay. *P <* *0.05, compared with MSCTRAIL/Control (Cont) growth on days 3 and 6, respectively, analysed by Student's *t*-test.

To determine the effect of cryopreservation on MSCTRAIL cell proliferation, morphology and differentiation, cells were defrosted and their stem cell characteristics assessed using flow cytometry for known MSC surface expression markers (CD73, CD90 and CD105) and differentiation into fat, bone and cartilage lineages. An XTT assay was used to determine whether cryopreservation altered the rate of cell proliferation. Notably, MSCTRAIL frozen in D5 solution exhibited significantly better proliferation potential than those cryopreserved with control freezing solution (Figure 2a). Expression of the characteristic MSC phenotypic markers CD105, CD90 and CD73 (Figure 2b) and lack of expression of the hematopoietic markers CD14, CD20, CD34 and CD45 (data not shown) showed no difference between D5 cryopreserved cells and routinely frozen counterparts in Cont solution. The differentiation potential of MSCTRAIL frozen in D5 solution was also examined and shown to be well preserved, with thawed cells capable of undergoing osteogenic, chondrogenic and adipogenic differentiation, respectively (Figure 2c). These data show that cryopreservation of MSCTRAIL in D5 solution does not affect MSC phenotype, proliferation and differentiation capacities.

### Cryopreserved MSCs maintain CXCR4 expression and cancer-homing ability

**Figure 3:**
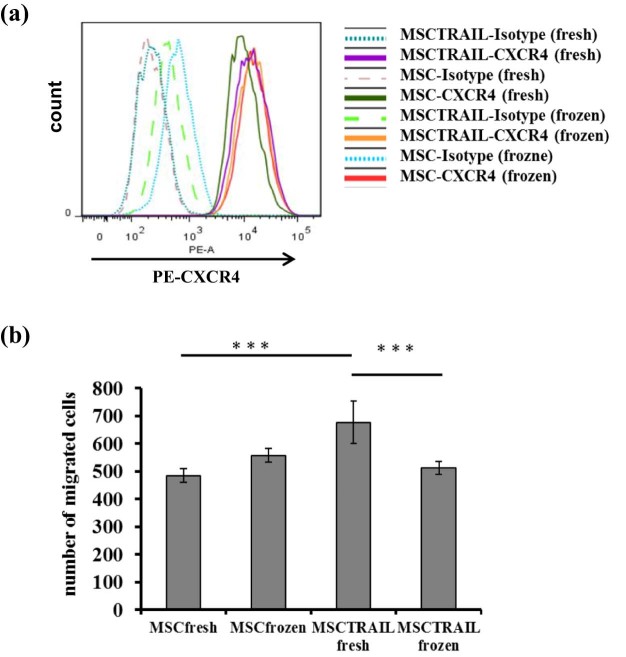
Analyses of CXCR4 expression and migration potential in fresh or cryopreserved MSC and MSCTRAIL cells (passage 5). (a) CXCR4 expression was measured by FACS. Cells were stained by an isotype immunoglobulin G-PE or by an anti-human-CXCR4-PE antibodies, respectively. (b) Three-dimensional collagen gel migration assay of fresh or cryopreserved MSCs and MSCTRAILs compared with fresh MSCTRAILs, respectively, analyzed by Bonferroni multiple comparison statistic test (***P <* *0.001).

The ability of MSCs to home to tumors is one of the key characteristics we are exploiting in our clinical therapy and one of the key mediators is CXCR4 [27]. To examine the effect of cryopreservation on CXCR4 expression in MSCs, we stained both fresh and thawed MSCs and MSCTRAIL cells with a PE-conjugated anti-human CXCR4 antibody and performed flow cytometry analyses. The cryopreserved cells showed equivalent CXCR4 expression levels compared with fresh cells, suggesting cryopreservation may not change MSC homing capacity (Figure 3a). To test this presumption, a three-dimensional collagen gel migration assay was performed. MSCs and MSCTRAIL, both fresh and defrosted, demonstrated good migration ability when chemo-attracted by cancer cell M231 conditioned medium (Figure 3b). Of note, fresh MSCTRAIL demonstrated significantly higher migration ability (migrated cell number 676 ± 77) than their thawed frozen counterparts (migrated number 512 ± 22), suggesting cryopreservation had affected MSCTRAIL homing potential although their CXCR4 expression not changed. However the effect appeared limited because the migration capacity of cryopreserved MSCTRAIL (512 ± 22) wascompatible with that of continuously cultured fresh MSCs (484 ± 24). This finding demonstrates that the tumor tropism of MSCs is maintained after cryopreservation in D5 solution, rationalizing the direct administration of thawed MSCTRAIL as a targeted anti-cancer therapy.

### Cryopreserved MSCTRAIL cells maintain TRAIL expression and effectively induce apoptosis in cancer cells

**Figure 4:**
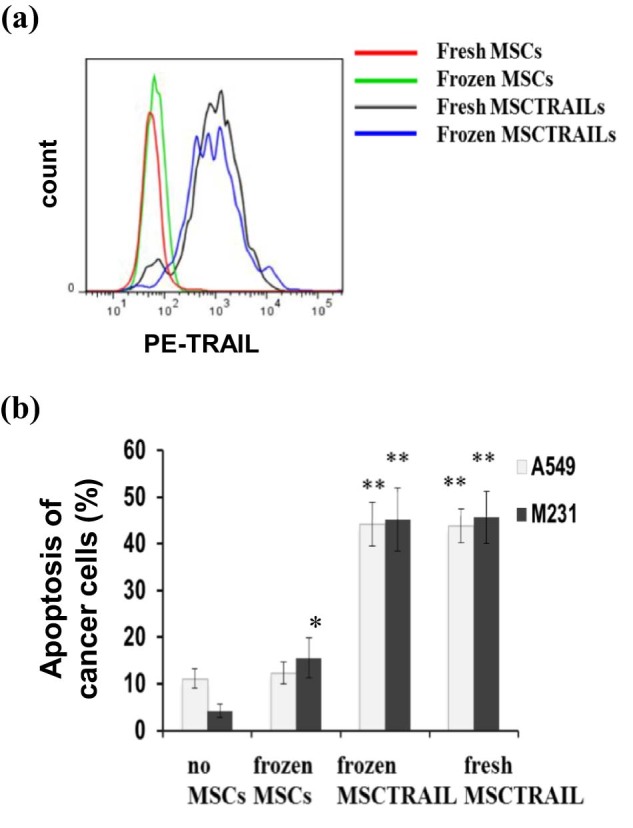
Cryopreserved MSCTRAIL maintain TRAIL expression and cancer cell–killing capacities. (a) Both fresh and post-thaw MSCs and MSCTRAILs (passage 5) were stained with a PE conjugated anti-human TRAIL antibody (ab47230) and analyzed by flow cytometry. (b). Fresh MSCTRAILs or post-thaw MSCs and MSCTRAILs were co-cultured with cancer cells A549 and M231 with a cell number ratio of 1:4 (MSC:cancer), respectively, followed by AF647-Annexin V antibody and DAPI staining for apoptosis assay. *P <* *0.05, **P <* *0.01 compared with no MSCs condition, respectively, analyzed by Student's *t*-test.

We next assessed cryopreserved and fresh MSCs for TRAIL expression and their therapeutic ability to induce apoptosis in cancer cells. Fresh and thawed MSCTRAIL cells were stained with a PE-anti-TRAIL antibody and analyzed by flow cytometry. There was no significant difference in TRAIL expression following cryopreservation (Figure 4a). To further confirm the biological activity of TRAIL in thawed cells, cryopreserved MSCTRAILs were defrosted and immediately added to DiI-labeled cancer cells at a ratio of 1:4 (MSCTRAILs: cancer cells) for overnight co-culture. For a comparison, fresh MSCTRAILs were also tested in parallel. All cells were stained and analyzed for apoptosis using annexin-V-AF647 and DAPI staining by flow cytometry and those that were DiI positive were assumed to be cancer cells. The breast adenocarcinoma line MDAMB231 (M231) and the lung adenocarcinoma line A549 were tested. Although cryopreserved MSCs showed no (A549) or low induction of apoptosis (M231) in cancer cells compared with no cell control (Figure 4b), both cryopreserved and fresh MSCTRAIL were equally effective in the killing of both M231 (45.2 ± 6.7% vs 45.6 ± 5.6% cancer cell death, respectively) and A549 cells (44.2 ± 4.7% and 43.9 ± 3.6%, respectively; Figure 4b). These observations show that MSCTRAIL cryopreserved in D5 solution retains its therapeutic efficacy.

## Discussion

This study is the first to look at the effect of cryopreservation on the homing ability of both transduced and untransduced MSCs and the stability of long-term genetic modification. We demonstrate that MSCTRAIL can be successfully cryopreserved in 5% DMSO with 95% ZENALB 4.5 without affecting cell viability, phenotype or differentiation potential. This is in line with previously published data, although in these studies, different cryopreservants were used, only some of which were clinically relevant [20], [28], [29]. Ten percent DMSO is associated with infusional toxicities in patients receiving cryopreserved cells, the severity of which is proportional to the amount of DMSO infused [26], so the ability to reduce the DMSO concentration used will have direct patient benefits. In addition, the removal of FBS from the formulation will be useful in meeting the current regulatory requirements for Good Manufacturing Practice process and improving safety for patients.

We have previously shown that intravenous delivery of MSCTRAIL eliminates and reduces lung metastases [7]. One of the critical steps in translating these research findings into a realistic therapy for patients is the development of a cryopreserved master cell bank that can be further expanded to produce a working cell bank that will be frozen before delivery to patients. The success of any treatment is reliant on cells maintaining their therapeutic properties after cryopreservation. The majority of clinical trials using cryopreserved MSCs have been in inflammatory conditions, including chronic obstructive lung disease [14], acute lung injury [30], graft-versus-host disease [15], [16], [17] and inflammatory bowel disease [31], with variable results. Thus, there have been a number of studies looking at the effect of cryopreservation on the secretory and immunomodulatory characteristics of MSCs, the results of which have been conflicting. Some studies show that cryopreserved cells have a reduced ability to suppress T-cell proliferation immediately post-thaw, which is fully restored after brief culture [21], [32], whereas others show that there is no effect [28], [33]. These differences may be explained by the use of different freezing media and the use of FBS during culturing. For those diseases that require the immunomodulatory properties of MSCs, it may be that cells must be cultured for a short time post-cryopreservation to have a therapeutic effect; with this step the cost and complexity of the manufacturing process increases, however.

As yet there is no potency assay for the immunomodulatory potential of MSCs, making it difficult to demonstrate whether cell potency and therefore therapeutic efficacy is affected by freezing. A recently published article comparing the ability of freshly thawed and continuously cultured MSCs to reduce allergic airway inflammation *in vivo* showed that the two products are equally effective [34]. Because our product delivers a therapeutic protein, we have an advantage as we are able to demonstrate effective induction of cancer cell apoptosis and death via a co-culture assay. We tested the ability of immediately thawed MSCTRAIL to kill two adenocarcinoma cell lines, A549 and M231, and showed that there was no reduction in TRAIL expression or the cancer cell–killing potency of our clinical product. Our phase 1/2a clinical trial is the first to deliver a genetically modified MSC therapy to lung cancer patients, and these results suggest that the production and cryopreservation of both a master and working cell bank is feasible without affecting the key characteristics required for therapeutic function.

One of the key features we are relying on in our therapy is the ability of MSCs to localize at the site of tumors. MSCs were first demonstrated as homing to distant sites when they were identified in the bone marrow of patients with osteogenesis imperfecta after systemic infusion. Not only did they home, but they were able to engraft and function resulting in increased growth velocity [35]. Subsequent studies looking at labeled MSCs suggested that intravenously delivered cells would be identified at high levels within the lung, liver and spleen but in low levels in other tissues [36], making the probability of true homing less likely. The most likely explanation for this is that MSCs accumulate in the pulmonary capillary system following intravenous delivery, hence their high levels soon after administration and interaction with adhesion molecules, such as vascular cell adhesion molecule (VCAM), which determines their biodistribution [37]. Tumors are thought to behave slightly differently and have been shown to be a target for injected MSCs [38]. Although the precise mechanism through which tumors attract MSCs is unclear, there is a consensus that CXCR4 plays a key role via its interaction with stromal cell–derived factor (SDF)-1 [39] and macrophage migratory inhibitory factor (MIF) [27]. Jarocha et al. [40] looked at the effect of pre-incubation with 10% DMSO on hematopoietic stem cells (HSCs) and showed a significant increase in the surface expression of CXCR4 with an associated enhanced chemotaxis to an SDF-1 gradient. This also correlated with increased *in vivo* homing to the bone marrow of sublethally irradiated mice [40]. However, this study only looked at the effect of DMSO without cryopreservation on a different cell type, thus the relevance of this work is not clear. Faint et al. investigated the effect of cryopreservation on lymphocyte migration and adhesion and demonstrated no functional effect but again noted an increased expression of CXCR4 [41].

Our data shows that in both transduced and untransduced MSCs, there is no change in CXCR4 expression following cryopreservation. Although this is not consistent with the previous studies [40], [41], it is likely due to experimental differences. We used a lower concentration of DMSO (5% compared with 10%), the previous studies both used pre-incubation of fresh cells rather than complete cryopreservation and subsequent thaw, and finally, they used different cell types. We saw a significant reduction in the number of migrating cells with cryopreserved MSCTRAIL compared with fresh counterparts; however, the difference is small, and levels of migration are still good and equivalent to untransduced cells.

Taken together, the results of our study demonstrate that both transduced and untransduced MSCs can be successfully cryopreserved in a combination of 5% DMSO and 95% ZENALB 4.5 without affecting cell viability or their immunophenotypic and differentiation properties. This is encouraging from a clinical perspective because it will reduce the incidence of infusion-related toxicities when delivering the cell therapy. We have determined a therapeutic window post-thaw that will enable us to accurately determine the duration of MSCTRAIL infusion without a loss of cell viability. We have also demonstrated for the first time that CXCR4 expression is not altered following cryopreservation in either transduced or untransduced cells and that although there is a reduction in cell migration, there is no reduction in TRAIL transgene expression or cancer cell killing efficacy. This suggests that we can safely produce a cryopreserved MSCTRAIL product that can be subsequently delivered to the patient in a clinical trial setting without a reduction in therapeutic efficacy and in a timely and cost-effective manner.

***Disclosure of interests:*** The authors have no commercial, proprietary, or financial interest in the products or companies described in this article.

## Figures and Tables

**Table I:** MSC freezing solutions.

Solution name	FBS (% v/v)	DMSO (% v/v)	α-MEM (% v/v)	4.5% HAS (% v/v)
Cont	30	5	65	0
D0	0	0	0	100.0
D0.5	0	0.5	0	99.5
D1	0	1.0	0	99.0
D2	0	2.0	0	98.0
D4	0	4.0	0	96.0
D5	0	5.0	0	95.0
D10	0	10.0	0	90.0
D15	0	15.0	0	85.0
D20	0	20	0	80.0

Cont, control freezing solution; D, DMSO; HAS, human albumin 4.5% solution (4.5% W/V solution for infusion human albumin solution; ZENALB 4.5, Bio Products Laboratory Limited).
